# BreathSense: A Two-Stage Digital Framework for Student Stress Monitoring Using Personalized Breath-VOC Thresholding and In-the-Wild Validation

**DOI:** 10.3390/bs16060934

**Published:** 2026-06-05

**Authors:** Anran Feng, Xingyu Zhao, Shengyu Gao, Cheryl Zhenyu Qian, Wanjun Li, Anping Cheng

**Affiliations:** 1 Patti & Rusty Rueff School of Design, Art, and Performance, Purdue University, West Lafayette, IN 47907, USA; qianz@purdue.edu; 2 Birmingham Institute of Fashion and Creative Art, Wuhan Textile University, Wuhan 430073, China; 3 Birmingham Institute of Fashion and Creative Art, Birmingham City University, Birmingham B4 7BD, UK; xingyu.zhao2@mail.bcu.ac.uk (X.Z.); shengyu.gao@mail.bcu.ac.uk (S.G.)

**Keywords:** mental health, exhaled volatile organic compounds (VOCs), stress monitoring, in-the-wild feasibility, personalized threshold

## Abstract

Student mental health and academic stress are increasingly addressed through digital monitoring, yet evidence for personalized physiological thresholds based on exhaled VOCs, their in-the-wild feasibility, and their trigger–experience correspondence in everyday student life remains limited. This study examines whether exhaled breath signals can support personalized, real-world stress monitoring in university students using a two-stage design that moves from laboratory calibration to daily life validation. A total of 24 university students took part in the laboratory phase (Study 1; N = 24). Under two stress tasks, a social-conflict video task and a Stroop task, we derived an individualized breath-trigger threshold (θ_i_) for each participant. We then invited 21 of them to join a three-day field deployment (Study 2; N = 21). Each participant’s θ_i_ from Study 1 was used directly as the trigger threshold for daily monitoring in order to test the association between trigger events and subjectively noticeable emotional deviations and to assess preliminary trigger–experience correspondence in daily life. The results show that 78.6% of paired trigger–EMA records were rated as subjectively salient, with 93.9% of these rated at medium-to-high intensity. These events occurred most frequently during study/work activities (60.6%), in dorm/home settings (57.6%), and when participants were alone (63.6%), suggesting that the triggers captured personally meaningful emotional episodes embedded in routine academic life rather than random physiological fluctuations. Overall, this study presents a portable breath-based emotion sampling device for student academic contexts and a reproducible protocol that combines laboratory thresholding with daily life validation. The findings provide preliminary and exploratory indications of the feasibility and within-person transferability of VOC-based emotion detection in students, and offer methodological support for future digital emotion monitoring and intervention design based on breath signals.

## 1. Introduction

Rates of anxiety, depression, and sleep problems among university students have been rising, leading to growing concerns about emotional health and, in turn, affecting learning performance and wellbeing (World Health Organization [WHO], 2025; Health Resources and Services Administration [HRSA], 2025). Prior research has shown that people’s emotional states can be inferred by detecting, interpreting, and modeling multiple nonverbal signals, such as facial expressions (e.g., raised eyebrows, smiling), vocal cues (e.g., pitch, rhythm), and bodily gestures (e.g., hand movements, posture) (Katsuyama et al., 2022; Schirmer & Adolphs, 2017; Busso & Narayanan, 2007; Babaei et al., 2020; Abdelrahman et al., 2015, 2017).

In recent years, olfaction has attracted increasing attention as a distinct and powerful channel for emotion communication. Odors can act directly on the limbic system, evoke deep memories, and subtly shape mood with little conscious awareness (Delplanque et al., 2017; Herz, 2000; Amores et al., 2018; Chang et al., 2025). Experimental evidence suggests that pleasant and unpleasant odors can rapidly modulate heart rate variability, cortical activation, and decision preferences (Delplanque et al., 2017; Ehrlichman & Halpern, 1988). Conversely, emotional states can also change body odor and breath chemistry. For example, anxiety may increase certain volatile compounds in exhaled breath, such as isoamyl alcohol and acetone, and others may identify emotional states through these VOC cues (Katsuyama et al., 2022).

These findings highlight a coupled relationship between olfaction and emotion and have motivated a growing body of work exploring breath and skin VOCs as noninvasive biomarkers of psychological stress and affective states (Lucchi et al., 2024). However, most existing studies focus on single laboratory tasks or specific clinical populations. Systematic evidence is still limited on whether individualized VOC thresholds, developed for university academic contexts, can transfer to daily life and reliably link trigger events with real-world emotional episodes.

Building on this gap, we developed BreathSense, a mobile breath-based emotion-monitoring approach with two components. The first component is portable breath-sampling and gas-sensing hardware that can be integrated with a smartphone to capture exhaled VOC signals in real time. The second component is an on-device software module that includes a 30 s “breath score” algorithm and an EMA interface triggered by this score, allowing users to view and log emotion-relevant events as they occur. Using BreathSense, the present study pursues three objectives: (1) to derive individualized breath-VOC trigger thresholds through controlled laboratory stress induction; (2) to examine whether these thresholds show preliminary trigger–experience correspondence when transferred to students’ daily academic lives over a three-day deployment; and (3) to descriptively characterize the everyday contexts in which trigger events occur as preliminary groundwork for future monitoring design. This work is intended for researchers in digital mental health, affective computing, and HCI-based physiological sensing who are exploring low-burden approaches to stress monitoring in student populations. To our knowledge, the combination of personalized breath-VOC thresholding and in-the-wild EMA validation within a single protocol has not previously been reported in everyday academic contexts.

## 2. Related Works

This section reviews the literature along two lines directly relevant to the present study. Section 2.1 examines the bidirectional relationship between olfaction and emotion, establishing the physiological basis for using exhaled VOCs as affective indicators. Section 2.2 then reviews practical challenges in everyday emotion measurement, including the shift toward individualized physiological sensing and the role of ecological momentary assessment (EMA) in real-world validation.

### 2.1. Olfaction and Emotion: Bidirectional Pathways to Breath-Based Sensing

Olfaction is often described as “the most emotional sense.” It can quickly evoke pleasant or unpleasant experiences, shift arousal toward alertness or relaxation, and trigger vivid affective memories (Roberts et al., 2022; Delplanque et al., 2017; Engen, 1982). Compared with other senses, olfaction has often been characterized as relatively elusive yet especially direct in its access to memory and emotion (Gibbons, 1986).

Across disciplines, evidence suggests a bidirectional influence of olfaction on emotion and behavior. On the one hand, the pathway from external odor input to emotional outcomes has been repeatedly supported in associative learning, motivated perception, and emotional memory, and odors can also modulate breathing patterns and subjective arousal (Herz, 2004; Homma & Masaoka, 2008). On the other hand, the reverse pathway also exists: emotional states and stress can shape breath composition. Psychological stress and metabolic load can lead to measurable shifts in exhaled VOC profiles, offering a chemical window into “emotion–metabolic echoes” (Miekisch et al., 2004). Beyond these core pathways, odors have been shown to influence the retrieval of emotional memories (Ehrlichman & Halpern, 1988), reduce anxiety through non-pharmacological interventions (Ballanger et al., 2019), and relate to emotional contagion processes (Freemantle et al., 2022). In human–computer interaction and wearable research, recent work has combined physiological monitoring with multisensory stimulation to modulate arousal and affect (Fekri Azgomi et al., 2023), while technology-probe studies suggest that affective responses to smells can be relatively consistent in daily contexts (Brianza et al., 2022). Together, this evidence supports two testable routes: from external odors to emotion and from emotion or stress to breath chemistry (VOCs).

### 2.2. Everyday Emotion Measurement: Individualized Sensing and Ecological Assessment

From both the classic body–mind tradition and modern neuroscience, emotion can be understood as an outcome of coupled bodily changes and subjective experience (James, 1894, 1922). Different “basic emotions” often show distinguishable family patterns in observable signals, preceding events, and physiological responses (Ekman, 1992). At the mechanistic level, sensory input can engage the thalamus–amygdala–prefrontal pathway to trigger defensive or approach responses and then cascade into activation of the autonomic nervous system (ANS) and the hypothalamic–pituitary–adrenal (HPA) axis. This, in turn, produces dynamic changes in breathing, heart rate variability (HRV), and metabolic activity (Critchley et al., 2004). This perception–emotion–physiology chain has been broadly supported in experimental work (Hutcherson et al., 2005; De Gelder, 2006) and provides the mechanistic basis for using peripheral metabolic signals, including exhaled VOCs, as potential indicators of affective states.

However, reliable emotion measurement in everyday life still faces practical constraints. Current approaches mainly rely on self-reports and wearable electrophysiological measures (e.g., heart rate/HRV and electrodermal activity), yet their stability, interpretability, and transferability across individuals and real-world settings remain under debate (Barradas et al., 2026). At the same time, recent wearable-based work has increasingly shifted from population-level models toward individualized representations and forecasting, learning person-specific temporal features that can support personalized mood, health, and stress prediction (Li & Sano, 2020). This trend underscores the practical value of individualized baselines and decision boundaries when transferring sensing systems from controlled settings to everyday life. Ecological momentary assessment (EMA) can capture daily fluctuations, but repeated reporting often leads to lower adherence and higher response burden. Differences in prompt frequency and timing can also substantially affect data quality and bias (Stinson et al., 2022; Portillo-Van Diest et al., 2024).

In contrast, evidence for breath chemistry as a potential metabolic window remains limited at the population level and in in-the-wild settings. Prior studies have mostly focused on small samples and controlled environments, with little systematic testing of individualized thresholds for “emotion/stress to VOC profile change”, daily life transferability and trigger–experience correspondence, such as time alignment and consistency with EMA reports. This gap is especially clear in adolescents and university students in everyday academic contexts (Miekisch et al., 2004; Lucchi et al., 2024; Mortazavi et al., 2025). In addition, although there is suggestive evidence that body odor, skin VOCs, and breath chemistry may play roles in emotion communication, threat cues, and emotional contagion (Mutic et al., 2016; Roberts et al., 2022; Katsuyama et al., 2022), it remains challenging to achieve reliable temporal alignment and diagnostic consistency within a mobile, low-burden, on-device, and interpretable framework.

To address these gaps, we adopted a two-stage research pathway. In Study 1, we used standardized induction tasks and modeled signals in 30 s windows, extracting individualized, channel-specific thresholds from induction-phase peak statistics (peak-based thresholding). In Study 2, we conducted a three-day in-the-wild deployment and aligned system triggers with EMA records to assess preliminary trigger–EMA correspondence in daily life. We also explicitly recorded proximal confounds such as activity, location, and social context to improve interpretability and external validity.

## 3. Overall Study Design for “BreathSense”

We initially recruited 36 students; 24 completed the laboratory phase (Study 1) and 21 subsequently completed the three-day field deployment (Study 2). Detailed inclusion and exclusion criteria and participant flow are reported in Section 4.2.2 and Section 5.2.1, respectively. All participants completed a resting baseline and two stress-induction tasks, and we derived an individualized trigger threshold (θ_i_) from their exhaled VOC signals. We then invited 24 eligible students from Study 1; 21 completed the three-day in-the-wild deployment (Study 2) and were included in the analyses. Three participants did not continue to Study 2 due to scheduling conflicts or voluntary withdrawal, with no indication of systematic differences from the remaining sample. Because each participant’s threshold was derived entirely from their own Study 1 data, the absence of these three individuals does not affect the thresholds or analyses of the 21 who completed Study 2, and the two studies’ analyses are statistically independent. Regarding sample size, the present study is designed as a feasibility investigation rather than a confirmatory trial, and the sample sizes of N = 24 (Study 1) and N = 21 (Study 2) are comparable to those reported in similar early-stage wearable VOC and physiological sensing studies (Lucchi et al., 2024; Plintz et al., 2025). In an individualized design, where each participant serves as their own reference, a smaller sample is sufficient to establish within-person threshold derivation and trigger–experience correspondence (see Section 6.4 for a discussion of sample size limitations).

In this phase, each participant’s θ_i_ from Study 1 was used directly as the trigger threshold for daily monitoring. We recorded threshold-triggered events together with EMA self-reports to examine the preliminary feasibility and trigger–experience correspondence of this threshold when moving from a controlled laboratory setting to real study and everyday life contexts, as shown in Table 1.

All study procedures were approved by the university ethics committee at Birmingham City University. Participants were screened at recruitment to ensure that they had no respiratory conditions, no known allergies to fragrances or chemical odors, no strong adverse reactions to smells, and were not pregnant. Before the study began, all participants were informed of the study purpose, procedures, and possible risks, and they provided written informed consent.

## 4. Study 1: Individualized VOC Threshold

### 4.1. Aim

In Study 1, our goal was to derive individualized, channel-specific trigger thresholds $\theta_{i,k}$ from four-channel exhaled VOC signals under controlled stress induction. We further examined whether these thresholds, together with changes in short-term signal activity, showed a clear induction-related shift from resting baseline to stress states.

### 4.2. Method

**Figure 1:**
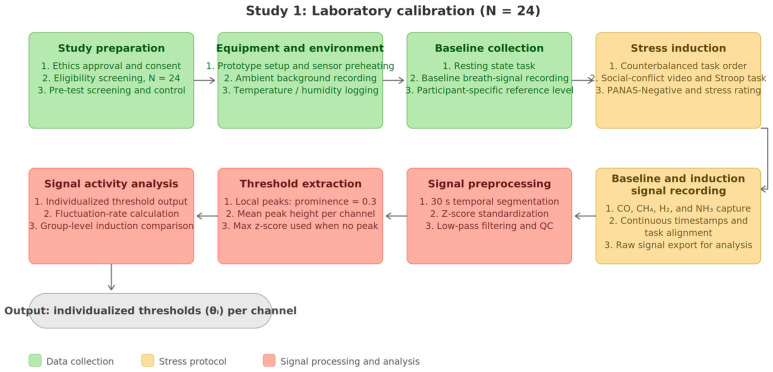
Experiment procedure of Study 1.

The Stage 1 experimental workflow, from study preparation and baseline collection to stress induction and signal processing, is summarized in Figure 1:

#### 4.2.1. Sampling and Data Quality Control

*Standardized sampling process.* We unified the sensor preheating time, sampling distance and posture, and sampling period to reduce systematic error caused by operational differences. Sampling conditions were also recorded to support reproducibility (Horváth et al., 2017).

*Environmental control.* We avoided strong VOC sources such as fragrances, detergents, and food. Before each acquisition, we recorded a background baseline of ambient gas to distinguish environmental fluctuations from expiratory changes.

*Temperature and humidity control and correction.* Temperature and humidity were recorded synchronously. During data processing, we considered their impact on the responses of MOX and other low-cost gas sensors. If needed, we applied regression-based correction or treated these variables as covariates (Abdullah et al., 2022).

*Drift and aging control.* Within the same testing stage, we used sensors from the same batch as much as possible. We followed a consistent preheating and baseline-correction strategy. In the analysis, we avoided making claims based on absolute values and instead focused on relative changes and trends as observable evidence (Vergara et al., 2012).

*Timestamp synchronization.* We ensured that the start and end times of stimulus materials/tasks, subjective labels, and sensor data were aligned on the same time base to avoid synchronization errors.

The BreathSense prototype integrates four MEMS gas-sensor channels (CO, CH_4_, H_2_, and NH_3_); the corresponding sensor modules and target gases are listed in Table 2.

#### 4.2.2. Participants and Ethics

Study 1 recruited 24 young participants (18–25 years, M = 21.3, SD = 2.2), all of whom were university or graduate students in good self-reported physical and mental health. Exclusion criteria were: (a) a history of respiratory disease or acute upper respiratory infection; (b) a known allergy to common environmental odors or fragrances; (c) a current smoker; (d) an impaired olfactory function; or (e) pregnancy. Of the 36 initially recruited participants, five were excluded due to smoking history, three due to acute upper respiratory infection at the time of testing, and four withdrew for personal reasons, leaving 24 participants included in Study 1 analyses.

#### 4.2.3. Procedure and Measures

Our protocol and system design follow prior work on odor-related affective responses and wearable sensing approaches for autonomic measurement (Ehrlichman & Halpern, 1988; Tonacci et al., 2021). To elicit emotional and stress responses, we used two established experimental tasks:

*Social-conflict video task.* We selected film clips containing tense social interactions, conflict, or negative events. Compared with neutral nature scenes, such clips can increase self-reported negative affect and physiological arousal (Gross & Levenson, 1995).

*Stroop conflict task.* Participants viewed stimuli in which a color word and the font color were incongruent (e.g., the word “red” displayed in blue) and were instructed to respond as quickly as possible based on the font color. Prior work shows that the Stroop task reliably induces cognitive load and psychological stress (MacLeod, 1991).

The order of the two tasks was counterbalanced using a 2 × 2 Latin square design (Keppel & Wickens, 2004). Half of the participants completed the video task first and then the Stroop task, whereas the other half completed them in the reverse order, to reduce order effects and fatigue-related confounds (Eiselmayer et al., 2020).

#### 4.2.4. Questionnaires and Measures

To capture subjective affect at key time points, we used the PANAS-Negative scale (Watson et al., 1988). After the tasks, participants also provided a 1–10 self-rated stress score. These subjective measures were used to compare against patterns in the breath score, although the primary goal of Study 1 was to derive individualized thresholds from the breath signals themselves.

### 4.3. Analysis and Results

#### 4.3.1. Preprocessing and z-Score Standardization

Continuous gas signals were segmented into non-overlapping 30 s intervals prior to standardization and peak detection, producing a fixed-granularity time series for each channel. For each participant *i* and gas-sensor channel *k*, we standardized the raw time series within each phase/segment to improve comparability across participants and channels. Specifically, given a raw signal $x_{i,k}(t)$, we computed the z-scored signal as:

$$z_{i,k}(t)=\frac{x_{i,k}(t)-\mu_{i,k}}{\sigma_{i,k}}$$

where $\mu_{i,k}$ and $\sigma_{i,k}$ are the mean and standard deviation of $x_{i,k}(t)$ within the same phase/segment. Following standardization, the z-scored signals were smoothed with a second-order Butterworth low-pass filter with a cutoff frequency set at 10% of the Nyquist frequency, suppressing high-frequency noise while preserving slower trends relevant to emotional reactivity. Environmental background signals were also recorded before each session as a quality check for ambient interference.

#### 4.3.2. Individualized Threshold Extraction

We derived a channel-specific individualized trigger threshold $\theta_{i,k}$ from the induction-period z-scored signal $z_{i,k}(t)$. We first identified local peaks in the induction segment. Peaks were detected using Python’s scipy.signal.find_peaks (Python version 3.13.5; SciPy version 1.17.0) with a minimum prominence threshold of 0.3, ensuring that only peaks clearly distinguishable from surrounding fluctuations were retained. Let the set of detected peak heights be:

$$p_{i,k}=\left\{p_{i,k}^{\left(1\right)},p_{i,k}^{\left(2\right)},...{,p}_{i,k}^{\left(n_{i,k}\right)}\right\}$$

where $n_{i,k}$ is the number of detected peaks.

We defined $\theta_{i,k}$ using the following rule:

$$\theta_{i,k}=\{\begin{array}{ll} \frac{1}{n_{i,k}}\sum_{j=1}^{n_{i,k}}p_{i,k}^{\left(j\right)} & ifn_{i,k}\geq 1\\ \underset{t}{\max}\;z_{i,k}(t), & ifn_{i,k}=0 \end{array}$$

When clear peaks were present $\theta_{i,k}$ reflected the participant’s typical peak level during induction. When peaks were not detected, we used the maximum z-score in that segment as a conservative alternative.

#### 4.3.3. Fluctuation Rate (FR)

To quantify how frequently short-term peaks occurred (i.e., signal “activity”), we defined the Fluctuation Rate for each participant and channel as:

$${FR}_{i,k}=\frac{n_{i,k}}{N_{i,k}}$$

where $n_{i,k}$ is the number of detected peaks and $N_{i,k}$ is the total number of sampled points in the segment. A higher ${FR}_{i,k}$ indicates more frequent short-term fluctuations in that channel.

#### 4.3.4. Group-Level Evidence of Induction-Related “Lift”

At the group level, stress induction produced a clear upward shift across all four channels. For each channel, participants’ induction thresholds $\theta_{i,k}$ were significantly greater than 0 (one-sample *t*-tests against 0 in z-score units; BH–FDR-corrected *p* < 0.05, with three of the four channels reaching *p* < 0.001), indicating that the social-conflict video and Stroop tasks elicited systematic increases in exhaled chemical signals rather than random fluctuations. Channel-specific descriptive statistics and inferential results are reported in Table 3.

Effect sizes varied by channel. Two channels (H_2_ and CO) showed the most robust induction effects (Cohen’s d = 1.718 and 1.581), suggesting higher sensitivity and greater consistency across individuals in the induction response, although absolute threshold values remained participant-specific. A third channel (NH_3_) showed a moderate-to-strong effect (d = 1.030). The remaining channel (CH_4_) (d = 0.554) also reached statistical significance but showed the largest between-participant variability (SD = 3.309).

##### Fluctuation Rate Increases Under Induction

Beyond threshold shifts, signal activity also increased during induction. Across all four channels, fluctuation rates ${FR}_{i,k}$ were significantly higher during the induction phase compared with baseline (paired tests *p* < 0.005; see Table 4 for channel-specific results), indicating that short-term peaks occurred more frequently under stress induction and that the signals became more “active” during emotional arousal. Effect sizes were medium-to-large across all channels (Cohen’s dz = 0.925–1.109).

#### 4.3.5. Individualized Thresholds and Signal Activity

Using the induction-phase signals, we obtained a set of individualized, channel-specific thresholds $\theta_{i,k}$ for all participants (N = 24, four channels each).

**Figure 2:**
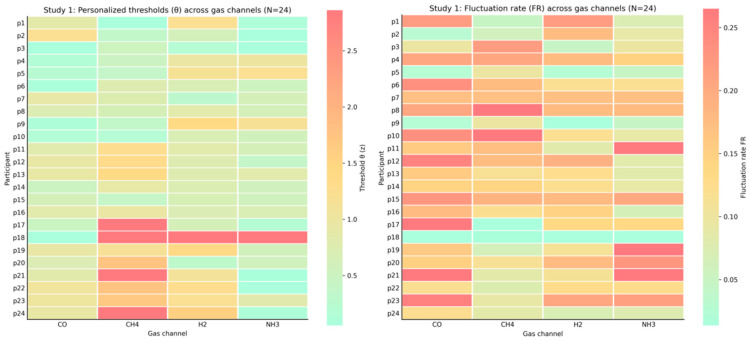
Participant-level calibration during induction. **Left**: thresholds; **right**: fluctuation rate.

Figure 2 visualizes participant-level calibration outcomes from the induction phase across four gas channels (CO, CH_4_, H_2_, NH_3_). The left heatmap reports the individualized trigger thresholds θ (z-score units) for each participant–channel pair, whereas the right heatmap reports the corresponding fluctuation rate (FR), i.e., how often the induction segment exceeded θ.

Thresholds (θ). The θ heatmap shows pronounced heterogeneity across participants and across channels within the same participant, supporting a “person × channel” calibration rather than a single universal cutoff. CH_4_ exhibits the largest between-participant dispersion, with several participants showing conspicuously high CH_4_ thresholds (e.g., p17, p18, p21, p24), indicating that larger CH_4_ deviations were required for those individuals to be flagged as salient. CO thresholds appear comparatively more clustered for most participants, while H_2_ and NH_3_ display selective elevations concentrated in a smaller subset of individuals. Notably, p18 shows concurrently elevated θ values across CH_4_, H_2_, and NH_3_, illustrating that conservative settings can be necessary in multiple channels for a single participant.

Fluctuation rate (FR). The FR heatmap demonstrates that supra-threshold activity is participant-specific and not dominated by one channel across the cohort. Elevated CH_4_ activity is most apparent for participants such as p8–p10, whereas NH_3_ activity is prominent for a different subset (e.g., p11 and p19–p21). CO shows higher FR for yet another group (e.g., p10, p17, p21, p23), while H_2_ tends to be moderate to high across many participants but still with clear individual variation. Importantly, θ and FR do not necessarily covary: a participant can present a high θ yet low FR (as for p18), consistent with fewer excursions surpassing a more conservative cutoff, whereas others show frequent crossings under moderate θ. Together, the two heatmaps indicate substantial individual differences in both calibrated sensitivity (θ) and realized channel activity (FR) during induction, motivating personalized multi-channel triggering for subsequent detection.

## 5. Study 2: In-the-Wild Feasibility Deployment over Three Days

### 5.1. Aim

The aim of Study 2 was to test whether the individualized channel thresholds $\theta_{i,k}$ derived in Study 1 can be used in daily life without further tuning. Using trigger-based ecological momentary assessment (EMA), we tested whether trigger events flagged by the ≥2-channel rule corresponded to subjectively salient deviations from each participant’s own daily baseline, and we described the intensity and contextual patterns of these EMA-labeled events over a three-day deployment.

### 5.2. Method

**Figure 3:**
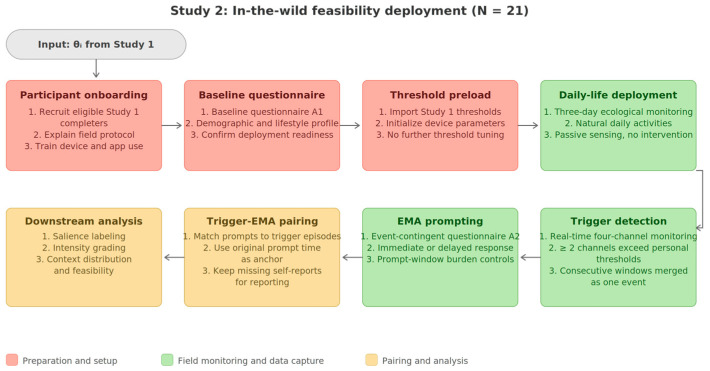
Experiment procedure of Study 2.

The Stage 2 in-the-wild workflow—including participant on boarding, threshold preloading, EMA prompting, trigger detection, trigger–EMA pairing, and downstream analyses—is summarized in Figure 3:

#### 5.2.1. Participants

Study 2 included 21 participants, all of whom had completed Study 1 and therefore had individualized thresholds $\theta_{i,k}$ for the four sensor channels. The inclusion and exclusion criteria followed Study 1 (e.g., non-smokers, normal olfaction, no known respiratory conditions or acute upper respiratory infection). Demographic and lifestyle information (e.g., year of study, daily routine, exercise and diet habits, and allergy profile) was collected through a baseline questionnaire to describe the sample and support interpretation of potential confounds.

#### 5.2.2. Setting

The in-the-wild observation lasted three consecutive days. Participants were not asked to change their schedules or locations and were encouraged to follow their usual routines. Typical contexts included dorm/home, study spaces (library/study rooms), classrooms, commuting, labs, and other campus public spaces. No intervention or feedback was provided. The system passively logged trigger events and EMA responses.

#### 5.2.3. Device and Triggering Rule (≥2 Channels)

**Figure 4:**
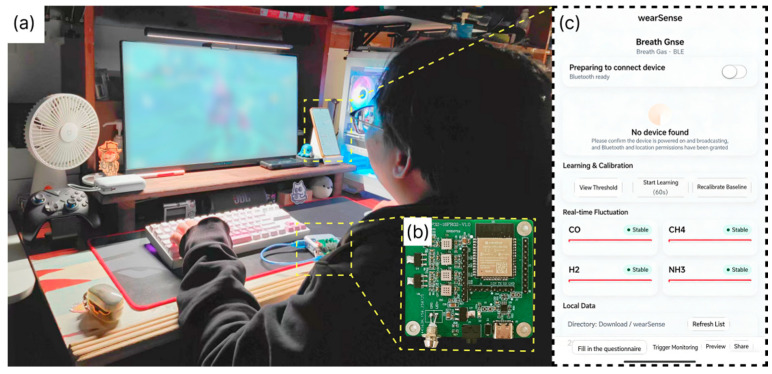
Hardware and software setup used in Study 2. (**a**) Example of in situ deployment during a participant’s everyday study context. (**b**) The custom BreathSense prototype integrating four gas-sensor channels for portable breath-VOC monitoring. (**c**) The companion mobile interface for device connection, calibration, threshold-based triggering, and real-time event logging.

Study 2 used the same BreathSense prototype as Study 1 (as shown in Figure 4). For each participant, the thresholds $\theta_{i,k}$ obtained from Study 1 were preloaded onto the device and used without further tuning. Sensor signals were standardized within each participant and channel using the same z-score procedure as Study 1, using the participant’s resting-baseline mean and standard deviation from Study 1 as the reference for z-scoring. A trigger event was logged when at least two channels met or exceeded their respective thresholds $\theta_{i,k}$:

$$\sum_{k=1}^{4}I\left(z_{i,k}\left(t\right)\geq \theta_{i,k}\right)\geq 2$$

where I(·) equals 1 when the condition holds and 0 otherwise, and $z_{i,k}\left(t\right)$ is the standardized signal for participant i in channel k at window t. Consecutive windows were merged and counted as one event.

#### 5.2.4. EMA Questionnaires

Two questionnaires were used.

***A1 (Baseline, Day 0):*** completed before deployment to collect demographics, study and sleep patterns, prior stress levels, and health/allergy background.

***A2 (EMA, Days 1–3):*** The A2 questionnaire included four 0–10 ratings: Tension (T), Pleasantness (P), Arousal (A), and Focus (F). The EMA also captured context (current activity, location, and social setting) and potential confounds in the prior 10–15 min (e.g., eating/drinking, coffee consumption, prolonged talking, wearing a mask, strenuous activity, use of fragrances/cosmetics). If participants could not respond immediately (e.g., while walking or in class), they could select “answer later,” which was recorded for later pairing. For the purposes of analysis, salience was defined from robust z-scored deviations on T, P, and A relative to each participant’s baseline, whereas intensity was graded using all four ratings (T, P, A, and F).

#### 5.2.5. Procedure and Prompting Rules

Participants completed the baseline questionnaire and received in-person training on how to use the device and the app, followed by a short trial run to ensure stable data collection. During Days 1–3, participants carried the device during their normal daily lives.

To reduce burden, EMA prompts followed three rules:

(1) prompts were delivered only between 08:00 and 22:30;
(2) no more than one prompt was delivered within any 15 min window;
(3) if triggers occurred frequently (e.g., three or more within two hours), a shortened EMA version was used (core ratings plus minimal context items).

After a trigger, participants could complete the EMA immediately or choose to respond later. The app issued one follow-up reminder; if the EMA was still not completed, the event was kept as “triggered but missing self-report” for response rate analysis.

#### 5.2.6. Pairing EMA with Trigger Events

If the participant answered later, pairing used the timestamp of the original prompt together with the completed response time to identify the corresponding trigger episode. For clusters of consecutive triggers, only the first trigger was treated as the event to be paired.

### 5.3. Analysis and Results

To evaluate trigger–experience correspondence, we analyzed paired trigger–EMA records in two steps. First, we applied salience labeling to determine whether each trigger corresponded to a subjectively salient deviation from the participant’s own short-term baseline (binary: salient vs. non-salient). Second, we applied intensity grading only to salient records to summarize the strength of these deviations as Low/Medium/High.

#### 5.3.1. Deployment Summary and EMA Completion

Over the three-day deployment, the system logged 46 trigger events and issued 46 EMA prompts. Participants completed 42 EMA questionnaires, yielding a response rate of 91.3%. Four trigger events had missing EMA responses after the reminder and were excluded from the paired analyses. The following results focus on the 42 paired trigger–EMA records. Two participants (ID 14 and ID 17) recorded zero trigger events during the observation window and therefore contributed no paired trigger–EMA records (their participant-level ratios are reported as NA).

#### 5.3.2. Salience Labeling Based on EMA (Binary)

To reduce between-participant differences in rating habits, we applied within-participant robust standardization to the four EMA ratings. Salience was then computed as the mean robust z-score across the three salience items (T, P, A). For each participant i and item j, robust z-scores were computed as:

$$z_{i,j=}\frac{x_{i,j}-median\left(x_{i,j}\right)}{1.4826\times MAD\left(x_{i,j}\right)}$$

where MAD is the median absolute deviation. If MAD = 0 for a given participant–item, we used a standard z-score based on that participant’s mean and standard deviation for the item. We then derived an unsigned composite deviation score by taking the absolute value of each item and averaging across the three items:

$${\left|z\right|}_{comp=}\frac{{|Z}_{T}|+|Z_{p}|+{|Z}_{A}|}{3}$$

where T, P, and A denote Tension (T), Pleasantness (P), Arousal (A), respectively. Binary salience decision rule. A paired trigger–EMA record was labeled as salient if either (i) any single item satisfied $|z_{i,j}|$ ≥ 0.9, or (ii) at least two items satisfied $|z_{i,j}|$ ≥ 0.6. Otherwise, it was labeled as non-salient.

#### 5.3.3. Trigger–EMA Alignment Results (Binary Salience)

Across the 42 paired trigger–EMA records, 33 were labeled as salient, yielding an overall alignment rate of 78.6% (33/42). This indicates that when the system triggered in daily life using the ≥2-channel rule and the individualized thresholds preloaded from Study 1, most trigger-aligned events corresponded to a clear self-reported deviation relative to each participant’s own short-term baseline. At the participant level, most individuals contributed only a small number of paired records over the three-day period (typically 1–4), and alignment varied across individuals, suggesting meaningful person-to-person differences in how VOC-based triggers map onto subjective experience in naturalistic contexts. Because the study did not include random or non-triggered EMA comparisons, this rate reflects trigger-event correspondence rather than diagnostic sensitivity or specificity; the present design supports preliminary feasibility but does not constitute a formal test of ecological validity in the measurement-theoretic sense.

#### 5.3.4. Intensity Grading of Salient Events (Low/Medium/High)

Intensity grading was applied only to records labeled as salient. After confirming salience, we quantified event intensity using the L2-norm composite deviation across the four EMA dimensions—tension (T), pleasantness (P), arousal (A), and focus (F):

$$S_{L2}=\sqrt{{{|Z}_{T}|}^{2}+{{|Z}_{P}|}^{2}+{{|Z}_{A}|}^{2}+{{|Z}_{F}|}^{2}}$$

Conceptually, $S_{L2}$ captures the resultant magnitude of multi-dimensional subjective change: events with larger deviations and/or deviations across more dimensions yield higher $S_{L2}$ scores. Based on predefined cutoffs, salient events were categorized as Low ($S_{L2}$ < 1.0), Medium (1.0 ≤ $S_{L2}$ < 1.8), or High ($S_{L2}$ ≥ 1.8).

As shown in Table 5, among the 33 salient events, 2 (6.1%) were classified as Low, 19 (57.6%) as Medium, and 12 (36.4%) as High. Overall, trigger-aligned deviations observed in daily life were predominantly moderate to high in subjective intensity, while low-intensity deviations were relatively rare. We additionally inspected how intensity varied across everyday contexts (activity and location) to support interpretation alongside the contextual summaries reported in the subsequent section. When examining intensity together with context, Medium and High events were observed across both study/work and leisure/social settings and in both dorm/home and classroom/office locations, indicating that higher-intensity deviations were not restricted to a single everyday context.

#### 5.3.5. Context Patterns of Salient Events

We summarized context distributions for salient events (N = 33) (Table 6). Salient events occurred most often during study/work activities (20/33, 60.6%), followed by leisure/social activities (10/33, 30.3%). Events were concentrated in dorm/home (19/33, 57.6%) and classroom/office (14/33, 42.4%). In terms of social setting, salient events most often occurred while alone (21/33, 63.6%), followed by remote/online interaction (8/33, 24.2%).

Although these results are descriptive and may partly mirror participants’ daily routines, they suggest that salient deviations were often experienced during individually sustained activities or low-density social situations, rather than in highly crowded or socially complex contexts. These distributions suggest that the context profile provides a grounded backdrop for interpreting in-the-wild triggers and underscores the importance of considering everyday context when linking breath-VOC deviations to subjective experience.

#### 5.3.6. Potential Confounds and Self-Reported Descriptors (Descriptive)

We summarized self-reported proximal factors for salient events (N = 33), including potential confounds in the prior 10–15 min and multi-select EMA descriptors of perceived reasons and bodily sensations (Table 7, Panels A/B/C).

***Panel A (potential confounds).*** Most salient events were not preceded by recent eating or drinking (93.9% reported “no”). Prolonged talking (≥5 min) was reported in 21.2% of salient events, and mask wearing was rare (3.0%). Overall, these descriptive results suggest that common short-term confounds were absent for most salient events, although extended talking may be relevant for a subset of events and should be considered when interpreting breath-related deviations.

***Panel B (perceived reasons; multi-select).*** Participants most frequently attributed salient events to academic/work pressure (20/33, 60.6%), followed by other/none (10/33, 30.3%) and time pressure (9/33, 27.3%). Less frequently endorsed reasons included rumination/worry (3/33, 9.1%) and social conflict (3/33, 9.1%), with environmental change and exercise/fast walking reported infrequently (1/33 each, 3.0%). In participants’ optional event notes, several leisure/social cases were described as gaming or e-sports episodes, during which participants reported high attention and tension despite the leisure label. Taken together, these descriptors suggest that salient deviations were most often interpreted within an academic/time-demand frame, while a smaller subset reflected interpersonal, situational, or engagement-related factors.

***Panel C (bodily sensations; multi-select).*** The most commonly reported sensations were faster heartbeat (22/33, 66.7%) and shortness of breath/rapid breathing (11/33, 33.3%). A notable minority reported no obvious bodily sensations (8/33, 24.2%), and dizziness/discomfort was uncommon (2/33, 6.1%). Overall, participants’ descriptors frequently involved autonomic-like sensations, but salient subjective deviations could also occur without strong perceived bodily cues. This underscores the value of pairing physiological sensing with brief self-reports for interpretation in naturalistic monitoring.

## 6. Discussion

This paper reports two connected studies evaluating whether individualized breath-VOC thresholds derived in the laboratory can support meaningful event prompting in daily life. In Study 1, we derived “person and channel” trigger thresholds from four-channel breath signals under standardized stress-induction tasks. At the group level, the induction phase produced a clear upward shift, while threshold magnitude and signal activity showed marked individual differences. In Study 2, we preloaded each participant’s threshold onto the device for a three-day in-the-wild deployment, applied a conservative ≥2-channel trigger rule, and assessed correspondence by pairing triggers with event-contingent EMA. Among paired trigger–EMA records, 78.6% (33/42) were labeled as salient deviations relative to each participant’s short-term baseline, offering preliminary exploratory indications of the feasibility of gas-based sensing as a complementary method for future stress monitoring. Descriptively, salient events were predominantly of medium-to-high intensity and tended to occur during study/work routines and indoor settings. Self-reports further indicated that events were most often attributed to academic/work pressure and commonly accompanied by sensations such as faster heartbeat and rapid breathing, whereas proximal confounds such as eating/drinking and mask wearing were infrequent (Picard, 2000; Fairclough, 2009; Calvo & D’Mello, 2010).

### 6.1. Discussion of the Two Studies

#### 6.1.1. Analysis of Non-Salient Triggers

In Study 2, 21.4% (9/42) of paired trigger–EMA records were labeled as non-salient. This outcome should not be interpreted as “wrong triggers” in a strict binary sense. In real-world deployments, there are several plausible but currently unverifiable reasons why a VOC-based trigger may occur without producing a strong deviation in the EMA ratings at that moment.

First, breath chemistry may reflect arousal-related physiology that is only partially captured by the selected self-report dimensions. Ventilation changes, metabolic shifts, and autonomic activation can affect VOC-related signals even when participants experience the moment as routine or do not appraise it as emotionally distinctive. Prior work suggests that acute cognitive stressors can shift exhaled VOC profiles (Turner et al., 2013). However, the present data cannot confirm whether this mechanism accounts for any specific non-salient trigger. In daily life, subtle workload transitions or effortful concentration may therefore produce physiological deviations that are not accompanied by a strong subjective change.

Second, temporal alignment in event-contingent EMA is inherently imperfect. Participants may respond with delays, multiple triggers may cluster within short intervals, and physiological excursions may be brief. EMA methodology emphasizes that feasibility and burden reduction often require compromises, which can introduce temporal mismatch and missingness (Doherty & Doherty, 2020). Event-contingent designs therefore focus on pragmatic correspondence rather than perfect synchronization. Within this framing, the overall alignment observed in this deployment remains informative as a preliminary indicator of trigger–experience correspondence.

Third, the meaning of “false positives” depends on the system goal. The present system is not intended for diagnosis; rather, it aims to support low-burden awareness and reflection. HCI work on stress/affect technologies emphasizes empowering users with gentle cues and avoiding clinical framing (Sanches et al., 2010). Under such a design goal, tolerating a minority of non-salient prompts may be acceptable—especially in early-stage prototypes—if it helps avoid missing meaningful events.

Fourth, residual behavioral and environmental factors may influence sensor responses without a strong subjective impact. Table 7 indicates that recent eating/drinking and mask wearing were infrequent around salient events, but daily life still includes micro-exposures and activity changes (e.g., prolonged talking, walking, ambient VOC fluctuations) that can affect low-cost sensors. Prior reviews highlight the impact of sampling context, temperature/humidity, and drift in field settings and recommend logging and correction strategies when moving from lab to real-life deployment (Tiele et al., 2020; Vajhadin et al., 2021).

Taken together, a non-salient proportion in this range is consistent with expectations for deployable physiological sensing: most triggers correspond to participant-perceived deviations, while a minority likely reflect subtle physiology, timing mismatch, or context-related sensor responsiveness. These remain interpretive possibilities rather than demonstrated explanations; the present dataset does not allow us to directly attribute non-salient triggers to any single mechanism. This pattern nonetheless supports iterative refinement rather than a negative interpretation of system performance.

#### 6.1.2. Analysis of Intensity Grading Among Salient Events

The intensity grading results add another way to interpret the salient events. Salience was defined relative to each participant’s short-term baseline, and intensity captured the magnitude of multi-dimensional EMA deviation. As a result, salient events tended to correspond to clear self-reported shifts rather than marginal fluctuations. In our data, salient events were mostly Medium or High in intensity, with Low being rare.

Three points help place this pattern in context. First, within-participant labeling emphasizes individual relevance: even in a non-clinical student sample, deviations can be meaningful when judged against a person’s own baseline. Second, EMA ratings can show range restriction and habituation over repeated prompts; therefore, fine differences in intensity in daily settings should be interpreted cautiously (Doherty & Doherty, 2020). Third, the descriptive context and self-reports suggest that salient events were often linked to academic/time-demand pressures and were commonly accompanied by bodily sensations such as faster heartbeat and rapid breathing. These descriptors make it plausible that many salient events were experienced as moderate-to-strong deviations.

Finally, activity labels do not always match subjective strain. In optional event notes, some leisure/social cases were described as gaming or e-sports episodes, during which participants reported high attention and tension despite the leisure label. This highlights a practical HCI point: the meaning of a physiological trigger depends on both bodily dynamics and how the person interprets the situation, not only on the activity category.

#### 6.1.3. Interpretive Scope of the Individualized Design

A feature of both stages that warrants explicit discussion is their fully within-person structure. Although stress induction produced a consistent directional shift at the group level across all four channels, the magnitude of response and the channel-specific pattern of reactivity varied substantially across individuals—a finding that motivates personalized thresholds rather than a single universal cutoff. In other words, the group-level evidence in Study 1 validates the sensing paradigm, while the individual-level variability justifies the calibration approach.

Two observations from Study 2 further illustrate the interpretive boundaries of this design. First, the two participants who recorded zero trigger events (ID 14 and ID 17) reported genuinely low emotional fluctuation during the deployment period in post-study feedback, rather than indicating that their thresholds were miscalibrated. This suggests that within-person threshold transfer can interact with individual differences in daily emotional reactivity in ways that are not predictable from the calibration session alone. Second, while some participants did trigger across different contexts—for instance, both in dorm settings and in classroom environments—the present dataset is too limited to draw conclusions about the within-person cross-context stability of the thresholds.

A more fundamental gap is that although basic demographic and lifestyle information was collected through the Study 2 baseline questionnaire, these variables were not systematically analyzed in relation to individual differences in baseline VOC levels and threshold magnitude. Participants such as p18, who showed concurrently elevated thresholds across CH_4_, H_2_, and NH_3_, illustrate that these person-level differences can be substantial and multi-channel, yet their sources remain opaque in the current data. Future work should incorporate structured background profiling alongside the calibration phase to begin disentangling physiological, behavioral, and contextual contributors to inter-individual threshold variability. Importantly, because both threshold derivation and salience evaluation are anchored to each participant’s own data, the findings speak to within-person calibration-to-deployment transfer rather than to population-level trigger norms; the observed alignment rate and threshold values should not be interpreted as generalizable cutoffs for uncalibrated individuals.

### 6.2. Discussion in Stress Monitoring

Recent stress-monitoring research has largely converged on wearable, continuous sensing paired with some form of ground truth labeling. A common pattern is a two-part pipeline: (i) physiological data collection using off-the-shelf or custom wearables, and (ii) ground-truth acquisition either from controlled stress induction in the lab or from free-living self-reports such as EMA (Li & Sano, 2020; Tazarv et al., 2021). In terms of data collection, prior stress-monitoring work has focused mainly on signals such as skin temperature, electrodermal activity/skin conductance, and environmental stressors. Chest-worn commercial devices can also provide more fine-grained measures, including ECG (Empatica, 2024; Garmin, 2024; Bent et al., 2020). Many biomedical studies further report customized systems designed to capture multi-channel data, such as electrodermal activity (EDA) (Subash et al., 2023; Shajari et al., 2023), skin temperature (Rachakonda et al., 2020), and electroencephalography (EEG) (Sheeraz et al., 2022; Lee et al., 2020). Whether to rely on off-the-shelf devices or to develop custom hardware typically depends on the target signals and the intended application context. Off-the-shelf wearables offer convenience and reproducibility, but they may lack required sensors, provide insufficient sampling rates, or otherwise fail to meet specific study needs. In such cases, researchers may combine multiple wearable devices or develop custom systems equipped with dedicated sensors (Akbulut et al., 2020; Rachakonda et al., 2020; Zubair & Yoon, 2020).

At the same time, even where EDA-, skin temperature-, and ECG-based approaches form relatively mature pathways for stress monitoring, their stability in real-world settings remains vulnerable to motion artifacts, variations in skin contact quality, between-person baseline differences, and non-specific fluctuations driven by changes in context (Smets et al., 2018; Pinge et al., 2024). This has motivated increasing interest in complementary channels beyond traditional physiological signals in order to broaden coverage and improve interpretability for stress and affective dynamics. Volatile organic compounds (VOCs), as chemical cues linked to metabolism, respiration, physiological activation, and environmental exposure, have emerged as a promising candidate modality. Prior studies suggest that exhaled breath and skin-emitted volatiles can exhibit measurable changes under psychological stress induction, indicating their potential as non-invasive inputs for real-time monitoring (Tonacci et al., 2019; Lucchi et al., 2024). At the same time, VOC-based stress research shows clear methodological and technological divergence: one line of work relies on laboratory-grade instruments such as GC–MS and PTR–MS to obtain chemically resolved evidence, whereas another employs low-cost multi-sensor “electronic nose” approaches to capture pattern-level changes that could support wearable or deployable systems but are more vulnerable to environmental interference, sensor drift, and strong inter-individual variability (Plintz et al., 2025).

Against this backdrop, as low-cost hardware matures and demand for wearable and deployable monitoring continues to grow, our two-stage studies provide preliminary exploratory indications of the feasibility of this approach. A system assembled from low-cost, multi-channel VOC sensing components not only captures measurable signal shifts under controlled laboratory induction but can also be used in everyday contexts to elicit event-contingent feedback that corresponds to subjective experience. In other words, this work supports the feasibility of VOCs as a deployable complementary modality within a lab-calibration and free-living validation pathway and highlights the practical potential of building and iterating prototypes using low-cost components.

### 6.3. Discussion of Future Studies

We outline several directions for future work, acknowledging that these proposals go beyond what the present data can directly support:

(A) Position breath/VOC sensing as a feasible, low-burden event-detection layer.

A growing body of work suggests that stress can be associated with distinguishable VOC-related patterns, including those observed in academic stress settings (Durán Acevedo et al., 2021) and broader stress-related volatilomics findings (Tonacci et al., 2019; Mansour et al., 2023). In this context, the present contribution is not chemical specificity or diagnosis, but rather the feasibility of a portable multi-channel setup with an interpretable triggering rule that can function under free-living conditions and support event-based sampling.

(B) Emphasize interpretability and closed-loop design rather than accuracy alone.

Affective and physiological computing research highlights that sensing systems become useful when they translate signals into user-facing meaning and support actionable feedback (Picard, 2000; Fairclough, 2009; Calvo & D’Mello, 2010). In our pipeline, the trigger rule is already interpretable (e.g., two or more channels exceeded a personal threshold), and EMA provides lightweight annotation. A practical next step is to strengthen explainability by linking triggers to short “why/what I felt” descriptors, showing simple before/after trends, and offering reflection prompts—while maintaining a non-diagnostic framing consistent with prior HCI guidance (Sanches et al., 2010).

(C) Reduce non-salient triggers through multimodal sensing or hybrid prompting strategies.

Many stress monitoring approaches improve robustness by combining multiple physiological channels (e.g., respiration features, HRV, GSR), and field studies demonstrate the feasibility of wearable-based stress monitoring in everyday contexts (Iqbal et al., 2022). For breath-based triggering, a pragmatic direction is either (i) adding a second modality to help disambiguate context-driven sensor fluctuations, or (ii) occasionally collecting non-triggered “baseline EMA” samples to estimate false-trigger rates and calibrate interpretation. This is aligned with mobile sensing and digital phenotyping approaches used in student populations (Wang et al., 2014), while preserving a potential privacy advantage compared with always-on audio or location sensing.

(D) Develop user-facing visualizations with an empowerment-not-diagnosis framing.

HCI work on stress/self-awareness tools emphasizes gentle feedback, reflection, and user control (Sanches et al., 2010). Related systems illustrate that simple and readable displays can support awareness and self-regulation without making clinical claims (McDuff et al., 2012; Snyder et al., 2015). The Study 2 descriptors (context, perceived reasons, and bodily sensations) provide preliminary indicators of what content might be relevant in such an interface; however, whether and how such displays would support user interpretation or behavior change remain open design questions that would require user studies to evaluate.

### 6.4. Limitations

The study still has some limitations:

First, the free-living deployment period was relatively short, covering only three days of daily routines, which limits our ability to evaluate the stability of individualized thresholds over longer timescales.

Second, the sample consisted primarily of university students, whose stressors and everyday contexts are relatively homogeneous; this constrains the generalizability of the findings to populations with different ages, occupations, or health conditions. Additionally, the sample sizes of 24 (Study 1) and 21 (Study 2) are appropriate for a feasibility study with an individualized design, but a larger sample would strengthen the stability of group-level effect estimates and allow more robust individual-level analyses.

Third, the chemical interpretability of the four-channel VOC-sensing approach remains limited.

Fourth, limitations inherent to EMA as a form of ground truth still apply. Although we used robust standardization and an event-contingent design to reduce bias, delayed responses, missing entries, and individual differences in subjective reporting may still account for some non-salient triggers.

Fifth, the study did not include random or non-triggered EMA samples alongside the trigger-based prompts. As a result, the present design cannot estimate false-negative rates, sensitivity, or specificity of the trigger rule, nor can it evaluate the incremental value of VOC-based triggering relative to ordinary time-contingent or random momentary sampling. Future deployments should incorporate a mixed-design EMA protocol—combining trigger-based and randomly timed prompts—to enable a fuller evaluation of detection performance.

Sixth, because both stages are fully individualized by design, the findings speak to within-person calibration-to-deployment transfer rather than population-level trigger norms; the interpretive scope of the individualized approach is discussed in detail in Section 6.1.3.

## 7. Conclusions

### 7.1. Main Conclusion

We conducted two studies to test whether individualized breath-VOC thresholds calibrated in the lab appear transferable to everyday monitoring without further tuning. Together, the results provide preliminary exploratory indications that a brief induction-based calibration may yield usable, interpretable trigger thresholds for in-the-wild, low-burden event prompting.

### 7.2. Theoretical Implications

This work contributes to the growing literature on personalized sensing for affective and stress-related monitoring in three ways.

First, it supports a person-centered view of physiological and chemical sensing. Rather than assuming a single universal threshold, our two-stage design treats baseline levels and reactivity as individual-specific and uses individualized decision boundaries to facilitate transfer from controlled calibration to everyday use. This perspective aligns with broader trends in digital phenotyping and adaptive modeling that emphasize individualized markers rather than population-level cutoffs (Sameh et al., 2024).

Second, the study contributes to emerging discussions on “breathomics” and wearable chemical sensing by illustrating a practical route from laboratory induction to real-world deployment. Recent reviews highlight both the promise of exhaled VOC sensing and the remaining translation gap, particularly the need for robust preprocessing, calibration, and context-aware interpretation outside the laboratory (Brinkman et al., 2024; Heng et al., 2025). Our findings provide empirical support that a lightweight individualized calibration can achieve meaningful correspondence in daily life when paired with EMA.

Third, our use of trigger-based EMA strengthens methodological links between passive sensing and in-the-moment self-report. EMA research increasingly distinguishes between signal-contingent and event-contingent designs and emphasizes that prompting strategies shape which experiences are captured in daily life (Wrzus & Neubauer, 2023). By using VOC-based triggers as event prompts and analyzing salience and intensity relative to each participant’s own baseline, this study offers a concrete template for applying sensor-driven event sampling to evaluate trigger–experience correspondence and reduce reliance on retrospective reporting (Delobelle et al., 2025; Stinson et al., 2022).

### 7.3. Practical Implications

The present findings are preliminary in scale—21 participants, a three-day deployment, and 42 paired records, with notable variation across individuals—and should be interpreted accordingly. Within these constraints, the results are nonetheless consistent with the feasibility of a deployable, low-burden workflow. This workflow combines a brief laboratory calibration to derive individualized thresholds, on-device preloading, conservative multi-channel triggering, and lightweight EMA prompts to support interpretation. This workflow is consistent with wearable stress-sensing recommendations that emphasize data quality, conservative decision policies, and the role of context in real-world wearable stress monitoring (Bolpagni et al., 2024; Vos et al., 2023).

Second, most salient events were rated as medium-to-high intensity, suggesting that BreathSense triggers may help identify episodes that participants experience as clearly noticeable deviations in everyday academic life. This includes moments that may be difficult to reconstruct through end-of-day recall. This has practical relevance as a starting point for designing context-aware reflection tools. However, translating these triggers into real-time support or early-warning applications would require substantially longer deployments, larger samples, and validation against independent ground truth measures, in addition to careful attention to ethics, user burden, and privacy (Delobelle et al., 2025; Wrzus & Neubauer, 2023).

Third, the contextual distributions observed in this study (e.g., study/work routines, indoor settings such as dorm/home and classroom/office, and frequently being alone) could motivate future iterations of trigger logic. For instance, the concentration of salient events in indoor study settings suggests that it may be worth exploring context-aware prompting policies, though such policies would need empirical validation before deployment. This approach aligns with broader mHealth sensing guidance that treats context as part of the decision policy rather than only a post hoc explanation (Stinson et al., 2022). For researchers and practitioners beyond the present sample, this study offers preliminary exploratory indications of the feasibility of a low-cost, two-stage protocol for transferring individualized breath-VOC thresholds from laboratory calibration to real-life monitoring. The protocol relies on standardized induction tasks and commercially available gas sensors rather than population-specific norms, which may facilitate adaptation across institutional and cultural contexts. However, breath-VOC baselines may vary across populations due to differences in diet, climate, indoor air quality, and lifestyle factors. Future studies should examine whether the calibration procedure transfers effectively to non-student populations and to settings beyond East Asian academic environments. Practitioners interested in adapting this approach should prioritize local pilot calibration and treat the present thresholds as person-specific rather than transferable defaults.

## Figures and Tables

**Table 1:** Summaries of the two studies.

Phase	Purpose	Setting	Data and Methods	Key Outputs
Study 1	Extract an emotion-trigger threshold (θ_i_) from exhaled VOC signals for each participant under controlled stress induction	Laboratory (social-conflict video + Stroop)	The on-device system continuously computed a breath score; four gas-signal channels were z-normalized to derive an individualized threshold	Each participant’s baseline VOC profile and an individualized threshold range for rest vs. stress
Study 2	Test whether these individualized thresholds show preliminary trigger–experience correspondence in daily life over 3 days	University students’ natural daily life contexts	EMA sampling; the device computed the breath score locally and logged above-threshold events (no intervention); momentary self-ratings were collected; subjective–objective salience was assessed	Each participant’s detection performance in real-world settings and the alignment between objective triggers and subjective salience

**Table 2:** Gas-sensor channels used in the BreathSense prototype.

No.	Sensor	Gas
1	Fermion: MEMS CO (GM-702B)	CO
2	Fermion: MEMS CH_4_ (GM-402B)	CH_4_
3	Fermion: MEMS H_2_ (GMV-2021B)	H_2_
4	Fermion: MEMS NH_3_ (GM-802B)	NH_3_

**Table 3:** Channel-specific descriptive statistics and one-sample *t*-test results for individualized thresholds (N = 24).

Channel	N	Mean θ	SD	Median	95% CI	*t*	df	*p* (FDR)	Cohen’s d
CO	24	0.625	0.395	0.766	[0.458, 0.792]	7.747	23	<0.001	1.581
CH_4_	24	1.833	3.309	0.938	[0.436, 3.230]	2.714	23	0.012	0.554
H_2_	24	0.961	0.559	0.812	[0.724, 1.197]	8.416	23	<0.001	1.718
NH_3_	24	0.614	0.596	0.579	[0.363, 0.866]	5.048	23	<0.001	1.030

**Table 4:** Paired-sample *t*-test results comparing fluctuation rates between baseline and induction phases (N = 24). All *p*-values are BH-FDR corrected.

Channel	Baseline FR (M ± SD)	Induction FR (M ± SD)	Mean Diff.	95% CI	*t* (23)	*p* (FDR)	Cohen’s dz
CO	0.018 ± 0.009	0.108 ± 0.097	0.090	[0.034, 0.146]	3.460	0.004	0.925
CH_4_	0.009 ± 0.006	0.119 ± 0.096	0.110	[0.053, 0.167]	4.147	0.002	1.108
H_2_	0.016 ± 0.010	0.091 ± 0.081	0.075	[0.028, 0.122]	3.474	0.004	0.928
NH_3_	0.015 ± 0.014	0.079 ± 0.058	0.065	[0.031, 0.099]	4.151	0.002	1.109

**Table 5:** Intensity distribution among salient events (N = 33).

Intensity	Count	Percent
Low	2	6.1%
Medium	19	57.6%
High	12	36.4%

**Table 6:** Context distribution of salient events (N = 33).

Variable	Category	Count	Percent
Activity	Study/Work	20	60.6%
	Leisure/Social	10	30.3%
	Other	2	6.1%
	Eating/Drinking	1	3.0%
Location	Dorm/Home	19	57.6%
	Classroom/Office	14	42.4%
	Vehicle/Transport	0	0.0%
Social setting	Alone	21	63.6%
	Remote/Online	8	24.2%
	With 1 person	4	12.1%
	With multiple people	0	0.0%

**Table 7:** Descriptive summary of salient events during the three-day deployment (N = 33).

**Panel A.** Potential confounds in the prior 10–15 min.				
**Confound Item**	**Yes (n)**	**Yes (%)**	**No (n)**	**No (%)**
Recent eating/drinking	2	6.1	31	93.9
Prolonged talking (≥5 min)	7	21.2	26	78.8
Wearing a mask	1	3.0	32	97.0
**Panel B.** Perceived reasons for the event (multi-select).				
**Reason**	**Selected (n)**		**Selected (%)**	
Academic/work pressure	20		60.6	
Other/none	10		30.3	
Time pressure	9		27.3	
Rumination/worry	3		9.1	
Social conflict	3		9.1	
Environment change	1		3.0	
Exercise/fast walking	1		3.0	
**Panel C.** Bodily sensations reported (multi-select).				
**Bodily sensation**	**Selected (n)**		**Selected (%)**	
Faster heartbeat	22		66.7	
Shortness of breath/rapid breathing	11		33.3	
No obvious bodily sensations	8		24.2	
Dizziness/discomfort	2		6.1	

## Data Availability

The data presented in this study are available on request from the corresponding author.
